# Editorial: Neonatal Brain Injury and the Search for New Therapies

**DOI:** 10.3389/fped.2022.933917

**Published:** 2022-06-09

**Authors:** Silvia Carloni, Francisco J. Álvarez, Daniel Alonso-Alconada

**Affiliations:** ^1^Department of Biomolecular Sciences, University of Urbino Carlo Bo, Urbino, Italy; ^2^Biocruces Bizkaia Health Research Institute, Cruces University Hospital, Barakaldo, Spain; ^3^Department of Cell Biology and Histology, School of Medicine and Nursing, University of the Basque Country (UPV/EHU), Leioa, Spain

**Keywords:** hypoxia-ischemia (HI), newborn, neonatal encephalopathy (NE), brain injury, therapies, neuroprotection

Hypoxia-ischemia is largely recognized as a major cause of brain injury in the perinatal period that can lead to neonatal encephalopathy (1, 2). When this occurs, it frequently leads to neonatal mortality or to severe long-term neurological deficits in newborns with devastating consequences both for the baby and its family, contributing to over 50 million disability-adjusted life years worldwide each year (3). Therefore, the study of the complex relationship among the different mechanisms/factors involved in the injury of the developing brain and the finding of new pharmacological approaches are a high priority in perinatal care. Accordingly, the characterization of peripheral markers would help to predict the development of putative damage, and their modulation after pharmacological treatments may anticipate its efficacy as a potential therapeutic intervention for translation to the clinical practice.

The Research Topic “*Neonatal Brain Injury and the Search for New Therapies*” includes novel and original contributions in the study of neonatal encephalopathy with the aim to give a comprehensive and groundbreaking overview of the pathophysiology and treatment of this disease. Original reports that explore and clarify the prognostic value of biomarkers and specific diagnostic interventions are included. This collection also highlights the most recent evidences of new targets for therapeutic intervention. In this Research Topic issue of Frontiers, we bring together a special collection of 15 articles contributing sound evidence for the key concepts outlined.

Some papers analyzed relevant peculiarities of preterm infants as a sensible population of patients at risk of brain injury. Ma et al. studied the dynamic functional connectivity in both term and late preterm infants and observed that the latter preferred to stay in a state with general weak connectivity between networks; authors also found that this preference declined as maturity increased. Shaw et al. highlighted that the poor long-term neurodevelopmental and behavioral outcomes observed in preterm births could be associated to an impaired oligodendrocyte development; authors concluded that promoting GABAergic action might improve outcomes. In an exhaustive review, Shandley et al. focused on the key role of nutrition for brain development in neonatal life, providing a wide-range synopsis regarding the role of nutritive and non-nutritive feeding in the neonate outcomes, the underlying mechanisms involved in neurophysiology, and the relationship of abnormal activity with brain injury in preterm and term infants. Terrin et al. evaluated how protein intake can affect the early cerebral growth in very low birth weight newborns, showing that several cerebral structures' measurements were affected by high protein intake when administered by parental nutrition, encouraging the administration mainly by enteral nutrition.

Three original studies focused on the important topic of the identification of prognostic factors that could correlate with the ongoing brain damage. Zheng et al. showed that perfusion magnetic resonance imaging could have an important role in the identification of hypoxic-ischemic encephalopathy, regardless of findings on conventional magnetic resonance imaging, and in the prediction of language and motor outcomes. Zhu et al., in addition, revealed that multi-slice radiomic analysis based on apparent diffusion coefficients metrics could provide more quantitative information on brain development in neonates with congenital heart diseases, suggesting that these measurements may be more clinically helpful to identify atypical brain development in patients. In an original study, Sweetman et al. investigated the connection between high cytokines levels observed after innate immune cell activation, brain injury and the outcome in infants with neonatal encephalopathy. Authors found that moderate or severe encephalopathy and mortality were associated with elevated interleukin-8 and granulocyte-macrophage-colony-stimulating-factor, pointing out that these cytokines may predict early outcomes in neonatal brain injury.

Several papers provided new evidence of neuroprotective strategies. Fernández et al. found that methylene blue, a guanylyl cyclase inhibitor with free-radical scavenger properties, reduced the retinal damage induced by perinatal asphyxia in the neonatal rat. Authors concluded that methylene blue could regulate key players of inflammation, matrix remodeling, gliosis and angiogenesis in the eye, whose treatment may prevent the deleterious visual consequences of perinatal asphyxia. Yu et al. demonstrated that the intranasal administration of exogenous interleukin-4 improved myelination and attenuated the functional deficits in a hypoxia-induced periventricular leukomalacia model. Kollareth et al. demonstrated that the acute injection of docosahexaenoic acid triglyceride emulsion provided a very similar protection as hypothermia in a neonatal mouse model of hypoxic-ischemic brain injury, indicating an advantageous treatment in providing a feasible and effective strategy in patients after hypoxia-ischemia injury. Purohit et al. demonstrated that the use of human cord blood derived from unrestricted somatic stem cells restored aquaporin channel expression, reduced inflammation and inhibited the development of hydrocephalus after experimentally induced perinatal intraventricular hemorrhage in rabbit. Kasala et al. analyzed the effects of the simultaneous use of morphine and caffeine on brain development. Authors revealed that the concurrent use of morphine, administered to premature neonates for pain control, and caffeine, used for apnea treatment, induced apoptosis and mitochondrial dysfunction in the developing brain compared to the individual use of the compounds. Chan et al. discussed in an appropriate review the links between the respiratory support of the preterm neonate and the brain injury patterns. Authors pointed that the use of animal models are essential resources for studying the pathophysiology of ventilation-induced brain injury, with important translational implications that can be helpful to outline the way to care preterm neonates, with the aim to improve their neurodevelopmental outcomes.

As of today, advances toward new neuroprotective interventions in hypoxic-ischemic encephalopathy have been limited by incomplete understanding of secondary processes. Alonso-Alconada et al. described that the subventricular zone could be affected by neonatal asphyxia. In their work, hypoxic-ischemic piglets showed a decrease in cellularity together with a reduction in both cell proliferation and neurogenesis in this neurogenic niche, suggesting that asphyxia could compromise the replacement of the lost neurons and the achievement of global repair. In a comprehensive review article, Kleuskens et al. provided an overview on the pathophysiology of cerebral hyperperfusion, commonly observed during the first 1–5 days in asphyctic neonates. Authors highlighted the gaps in current understanding in term animals and neonates, analyzing data from both the hemodynamic changes and the endogenous pathways involved. They concluded that these findings should be simultaneously considered together with the brain imagining techniques, becoming a valuable resource in assessing the impact in neurodevelopmental outcome.

In summary, this Research Topic provides original articles and reviews that, together collected, may add new information on the epidemiology, pathophysiology, diagnosis and management of brain injury in the neonate, also identifying relevant treatments for testing in future clinical trials.

## Author Contributions

SC wrote the draft. FÁ and DA-A critically reviewed the manuscript. All authors contributed to the article and approved the submitted version.

## Funding

DA-A was supported by EITB Maratoia-BIOEF (BIO18/IC/003) and the Spanish Ministry of Science and Innovation (MINECOR20/P66/AEI/10.13039/501100011033).

## Conflict of Interest

The authors declare that the research was conducted in the absence of any commercial or financial relationships that could be construed as a potential conflict of interest.

## Publisher's Note

All claims expressed in this article are solely those of the authors and do not necessarily represent those of their affiliated organizations, or those of the publisher, the editors and the reviewers. Any product that may be evaluated in this article, or claim that may be made by its manufacturer, is not guaranteed or endorsed by the publisher.

## References

[1] Gunn AJ Thoresen M. Neonatal encephalopathy and hypoxic-ischemic encephalopathy. Handb Clin Neurol. (2019) 162:217–37. 10.1016/B978-0-444-64029-1.00010-2 31324312

[2] Volpe JJ. Neonatal encephalopathy: an inadequate term for hypoxic-ischemic encephalopathy. Ann Neurol. (2012) 72:156–66. 10.1002/ana.23647 22926849

[3] Lee AC Kozuki N Blencowe H Vos T Bahalim A Darmstadt GL. Intrapartum-related neonatal encephalopathy incidence and impairment at regional and global levels for 2010 with trends from 1990. Pediatr Res. (2013) 74(Suppl 1):50–72. 10.1038/pr.2013.206 24366463 PMC3873711

